# Workplace Discrimination Against Pregnant and Postpartum Employees: Links to Well-Being

**DOI:** 10.3390/ijerph22081160

**Published:** 2025-07-22

**Authors:** Kimberly T. Schneider, Sarah C. Williams, Rory E. Kuhn

**Affiliations:** Department of Psychology, Illinois State University, Normal, IL 61761, USA; scwill4@ilstu.edu (S.C.W.); vekuhn@ilstu.edu (R.E.K.)

**Keywords:** pregnancy discrimination, postpartum experiences at work, working mothers, breastfeeding, intersectional identities and work, social support

## Abstract

Pregnancy-related discrimination at work is a concern for many employees who navigate the pregnancy and postpartum stages of parenthood while working in the early-to-middle stages of their careers. Although there is legislation prohibiting pregnancy-related discrimination and ensuring accommodations postpartum, empirical evidence indicates many pregnant and postpartum employees still experience such behaviors. In this narrative review, we focus on describing the range of behaviors assessed in studies on pregnancy-related discrimination in several cultures, situating the occurrence of discrimination within theoretical frameworks related to stereotypes and gendered expectations. We also review evidence of employees’ postpartum experiences with a focus on the transition back to work, along with breastfeeding challenges related to pumping and storing milk at work. Regarding coping with pregnancy-related workplace discrimination and postpartum challenges during a return to work, we review the importance of social support, including instrumental and emotional support from allies and role models.

## 1. Introduction

For many employees, pregnancy and postpartum phases occur simultaneously with early-to-middle career years in the workforce, impacting their work experiences and career progression [1]. In this narrative review of recent research on workplace pregnancy-related discrimination across cultures, we focus on presenting a broad perspective of recent empirical research from the past 30 years to describe and synthesize employees’ experiences at work with reference to the relevant theory described below. Legal protections in the U.S. implemented in 2023 are relevant to nursing employees and may soon impact their experiences with breastfeeding discrimination; this is also described in a section below. We review empirical research on employees’ experiences with pregnancy and postpartum workplace discrimination, impact on employees’ well-being, and links to social support as effective coping responses. Whereas many of the studies reviewed here focused on descriptive indicators of employees’ experiences, we also highlight evidence of potential responses and interventions that have promising results in reducing discrimination using legal reforms and policy change.

Both sex role theory [2] and social role theory [3] are applicable to instances of pregnancy-related discrimination and predict that pregnant and postpartum employees are often impacted by gender stereotypes and role-related expectations in patriarchal cultures [4] and gender-neutral cultures [5]. Pregnant employees, those dealing with infertility challenges, and even those who choose not to be pregnant are affected by coworkers’ and supervisors’ prejudices stemming from gendered stereotypes of parenthood as well as work-related expectations of effective employees. Additionally, human capital theory emphasizes the work-related consequences of parenthood decisions for women, such as fewer career opportunities, wage penalties, and work-family conflict tied to division of labor [6]. These effects will be discussed in more detail in the sections below.

Globally, many employees are legally protected from discrimination related to pregnancy and pregnancy-related conditions. In the U.S., the Pregnancy Discrimination Act in 1978 amended Title VII of the 1964 Civil Rights Act to include protections for women who are or might become pregnant. Additionally, the Pregnant Workers Fairness Act of 2023 requires employers to make reasonable accommodations for employees based on any pregnancy- or childbirth-related condition, and the PUMP for Nursing Mothers Act includes protections for breastfeeding employees [7]. Several other countries have similar laws prohibiting discrimination, including the Equality Act in the United Kingdom [8]. Despite federal regulations that prohibit discrimination, empirical work and legal records indicate that employees do experience and report cases of pregnancy-related and postpartum discrimination. Many working women (We note that employees with other identities aside from “women” may become pregnant; most empirical research uses the female pronoun in describing their results, and thus in our summary of that research, we will reference “women” for consistency.) will experience pregnancy at some point during their careers [9].

In this narrative review, we describe research on employees’ experiences during pregnancy and postpartum phases, as well as the workplace context and challenges related to breastfeeding and pumping at work. Where research exists, we describe issues related to intersectional identities, as well as the ameliorating impact of support on employees’ stress. We examine research from various fields, including organizational psychology, clinical psychology, gender studies, medical research, legal perspectives, and large-scale sociological studies.

## 2. Methodology

We used the narrative review guidelines described by Green et al. [10] to structure the process of searching and filtering empirical articles. We used the following databases and search engines to begin our search: Web of Science, Scopus, Sage, PubMed, PsycInfo, and Google Scholar. Using the search terms “pregnancy-related discrimination at work”, “postpartum discrimination at work”, “breastfeeding discrimination at work”, and “pumping at work”, we searched for empirical articles and books published between 1994 and 5 January 2024. This resulted in a total of 68 empirical journal articles and books published in the English language. Upon reading and summarizing these relevant articles, we also searched the reference sections for additional work and added 14 citations. We summarized and synthesized this work, and we describe it in the sections below.

Although we focus on the experiences of pregnant or antepartum and postpartum working women in this review, we also note, but do not include due to space limitations, research focused on employees who must cope with infertility issues [11] and those who miscarry, including how stigma and taboos in some cultures may impact both pregnant employees and their partners (see [1] for an excellent review). We also note the existence of important research on employees who adopt children and the associated work-family conflict tied to that transition [12,13], as well as research on discrimination experienced by pregnant applicants in the hiring stage [14,15].

## 3. Literature Review Topics and Summaries

### 3.1. Pregnant Employees and Workplace Discrimination: Antepartum Phase

Across many cultures, women who work part- or full-time jobs while pregnant (i.e., antepartum) are impacted by gendered expectations and sex role stereotypes that influence their interactions with coworkers and supervisors. Grandey et al. argued that all working women are impacted by maternity, either through direct experiences or generalized expectations around parenthood [1]. Work-related discrimination and biases related to employees’ pregnancies are often prompted by stereotypes of the “ideal employee” [16] and are considered a type of gender-based discrimination. Pregnant employees may first encounter threats due to these stereotypes when determining the timing of when to disclose a pregnancy at work [1]. Stereotypes of high-performing employees in many cultures often focus on the dedication of time and energy to the organization, as well as commitment to coworkers. These expectations may conflict with stereotypical views of pregnant women as cognitively incompetent (“pregnancy brain” [17]) or physically weak [18]. Postpartum women also face interactions at work with those who hold stereotypes of working mothers being easily distracted by family issues, along with challenges related to breastfeeding upon returning to work (the latter of which is described in more detail below).

Sabat et al. discussed the decision that pregnant employees must make regarding when and how to disclose a pregnancy to supervisors and coworkers [17]. They likened pregnancy to other concealable stigmas, although it differs in that, over time, most pregnancies will become visible to others. In a Dutch sample, the presence of other pregnant coworkers who were role models reduced the chances that pregnant employees would conceal their pregnancies during the first trimester [19]. Some supervisors may have the perception that an employee’s pregnancy will involve disruptions in the work team due to the need to take time off, with others also assuming that the employee may become less committed to work [17]. Pregnant employees may have concerns regarding coworkers’ questions about their job dedication and performance [20]. Interviewing employees who were pregnant or new mothers, Millward described how women felt systematically excluded, felt invisible, and felt pressured to work harder due to perceptions of being a burden to coworkers [21]. Women in that sample also reported feelings of guilt stemming from these prejudicial views of their coworkers. Sabat et al. focused on disclosure versus concealment of a pregnancy in sample of pregnant employees and a separate sample of those who had experience supervising a pregnant employee. In both samples, trust in one’s supervisor, as well as anticipatory and realistic concerns about discrimination, were related to concealing a pregnancy until the late stages [17]. Based on research evidence, across many cultures, it is understandable why employees may be reluctant to disclose pregnancies, as they may be concerned that they will subsequently be at risk of being reassigned to riskier work or even fired, regardless of whether their country or region has anti-discrimination laws [22].

### 3.2. Assessment and Experiences of Pregnancy-Related Discrimination at Work

Regarding the assessment of specific discriminatory behaviors experienced by pregnant employees, there is variation in researchers’ approaches to this measurement. For instance, some researchers use measures of gender-based or race-based discrimination that are revised to reference pregnancy [23] or that are developed based on a particular country’s legal guidelines prohibiting discrimination [24]. One example of the latter approach to item development focused on discriminatory behaviors such as social isolation because of one’s pregnancy, receiving fewer opportunities at work, having one’s work checked more closely, and being discouraged from aiming higher in one’s career. In a longitudinal sample of pregnant working women in Japan who were surveyed both during pregnancy and at two months postpartum, Kachi et al. [24] reported that nearly 24% of their sample experienced at least 1 of the 16 discriminatory behaviors listed in their inventory. This was similar to the prevalence rate found by the same authors in an earlier cross-sectional study in Japan [25]. In the longitudinal sample, women who experienced discrimination were more likely to have lower education levels, work part-time, and work at smaller companies. These women were also more likely to report higher levels of postpartum depressive symptoms than those who did not experience pregnancy-related discrimination.

To capture a range of potential discriminatory behaviors, Borrowman et al. developed the Perceived Occupational Pregnancy Discrimination Questionnaire [26]. This self-reporting measure includes items ranging from being physically harassed (e.g., “forced to stand” or “someone smoking nearby”) to experiencing pay cuts and being forced to resign. They reported that the most common experiences in their sample of U.S. employees in their third trimesters included missing out on training and being treated poorly. Similarly, in a sample of British workers in their second and third trimesters, the most common forms of pregnancy-related discrimination included being given unreasonable workloads, being encouraged to take time off, or being encouraged to start maternity leave early [23].

Using an interview approach to gather data on the experiences of pregnant working women, Hennekam reported that women described external pressure and uninvited advice on balancing work and non-work, especially among those working in female-dominated sectors such as education [27]. As the sample was Dutch, the findings may have partly been a function of cultural norms for women to move to part-time work after having children. Using an intersectional approach in interviewing Black women working in retail, education, healthcare, and government jobs in the U.S., Mehra et al. described how women felt they were under scrutiny at work daily, were treated as outsiders, and had coworkers question their competence and performance [15]. In many cases, the resulting stress prompted women to either not return to work postpartum or find a new job.

It is notable that legal indicators in the U.S. show relatively stable levels of pregnancy-related discrimination claims filed with the Equal Employment Opportunity Commission from 2010 to 2024 [28]. In 2024, most pregnancy-related discrimination charges were related to firings (around 80% of complaints), followed by reassignments (73%), harassment (32%), terms and conditions of employment (25%), and retaliation (18%). As evident from the EEOC data, it is likely that employees experiencing discrimination are targeted in multiple ways (e.g., both reassignment and harassment).

### 3.3. Links Between Pregnancy and Postpartum Discrimination and Mental Health

These reports prompt concern related to the direct and indirect effects of working daily in such environments on women’s mental health and well-being. There are links between self-reported psychological stress at work and pregnant women’s mental health [29], as well as adverse birth outcomes, such as lower weight, as a correlate of job strain [30]. In two samples of full-time working women, Hackney et al. described the links between higher incidence of perceived pregnancy discrimination and higher levels of postpartum depression, lower birth weights, and increased doctor visits for babies [23].

In a longitudinal study on pregnant working women in the U.S. assessed in their third trimester and again six weeks postpartum, 43% of the women who experienced pregnancy discrimination reported scores greater than or equal to the clinical threshold for depression [26]. Similarly, 33% of the targeted women in that same sample reported scores indicating clinically significant anxiety symptoms. This provides additional evidence that the impact of discrimination on women’s postpartum symptoms is likely to occur through the experience of stress [23,26]. These negative impacts on women’s mental health could be ameliorated by higher levels of social support, however. Borrowman et al. observed that social support is modifiable, as some women in their sample who reported low levels of social support at the beginning of pregnancy were able to increase support over time [26].

Pregnant employees’ experiences of pregnancy and postpartum discrimination may focus on questions regarding work performance and commitment and may have impacts on salaries, with the “motherhood wage gap” approaching 4%, as noted in a recent meta-analysis [31]. Financial concerns are often precursors to employee stress. In a UK sample of female academics, concerns related to financial support during maternity leave were more likely in situations where universities had ambiguous policies and decision making regarding eligibility for paid leave [32]. The type of contract the pregnant employee worked under was highly predictive of her decision to return to work postpartum, with those holding fixed-term contracts less likely to return to work than those with open-ended contracts [32].

### 3.4. Vulnerability to Discrimination Based on Occupation and Intersectional Identities

Many of these issues described above relate to working pregnant women feeling uncertain or vulnerable regarding their work status, support from others on the job, or job security. Both financial and psychological stress may result from pregnancy-related discrimination [15], particularly for pregnant workers who are single. Financial stressors related to job security can also expand to include concerns related to health benefits and insurance. Working women who are in financially risky situations may be unwilling to leave if they experience discrimination or harassment, remaining in a job context where they experience high levels of stress. Psychological stress is evidenced in women’s reports of feeling depressed that their job is at risk, feeling angry or frustrated by not being offered opportunities at work due to pregnancy, or being restricted from taking on certain tasks [15]. Stress reactions to pregnancy-related discrimination may be particularly impactful on the mental health of those who do not have support systems in place [20].

It is also important to highlight that women who are minoritized based on ethnicity, race, religion, national origin, or being in a male-dominated occupation report feeling even higher levels of vulnerability, both objectively and subjectively. Borrowman’s longitudinal sample of pregnant and later postpartum women who experienced ethnic discrimination had increased odds of depression and anxiety (10% and 17% higher, respectively) than those who did not experience ethnic discrimination [26]. A 2023 analysis of race, motherhood, and economic data by the National Partnership for Women and Families in the U.S. determined that Black women who had recently given birth were significantly more likely to be economically insecure, defined as living in a family below 200% of the federal poverty line. In the U.S., Black working women represented a disproportionate share of pregnancy discrimination claims filed from 2011 to 2015. The most frequent claims included being fired for taking maternity leave, enduring extreme levels of manual labor, and being denied promotions and raises [33]. This highlights the need for the intersectionality of women’s identities to be acknowledged in studies on pregnancy and postpartum experiences.

Vulnerability to pregnancy-related discrimination is also magnified when employees from minoritized groups are working in high-risk, physically demanding jobs. Women of color and women who are immigrants are a disproportionate share of employees in these positions. For instance, in a 2014 report, the National Latina Institute for Reproductive Health reported data from the U.S. workforce indicating that Latina women made up 26.1% of employees who were hand packers, who are often required to be on their feet all day, loading and lifting heavy objects. Black women in the U.S. made up 28.4% of home healthcare workers, who spend time lifting elderly clients and cleaning. Immigrant women made up 44.9% of home cleaners, often spending most of the day walking and standing, in addition to being exposed to potential contaminants [34].

Much of the research related to women’s pregnancy experiences includes samples of working women who work regular shifts during daytime hours. There is a need for additional studies that focus on women in various occupational roles, including precarious work, part-time work, self-employment, and gig work. Jobs that take a physical toll on a pregnant employee put them at a higher risk of preeclampsia, diabetes, and hypertension, with evidence that these effects are even more impactful for those working irregular or night shifts [35]. Work conditions can negatively impact mental or physical health due to safety risks (i.e., exposure to disease or falls, relentless physical labor, or standing for long periods), and thus variety in terms of work settings, shifts, and work roles is needed in empirical research. Salihu et al. noted that pregnant employees who must mitigate chemical and biological risks are understudied, and the few samples with employees working in those conditions are drawn from the U.S., UK, Canada, or European Union [36]. Employees not only face potential mental and physical health risks while pregnant, but they may also have similar experiences upon their return to work postpartum.

### 3.5. The Postpartum Stage, Work Performance, and Well-Being

The postpartum return-to-work transition is a potentially stress-inducing period wherein new parents must navigate details related to childcare, work-family conflict, and work demands, all of which are influenced by individual, organizational, and cultural variables [37].

In general, the postpartum stage is a time of new routines and potential changes in relationships with people at work, especially for first-time mothers who may be navigating identity issues as various family-related and work-related roles come into conflict. Role conflict theories postulate that employees will experience stress when they perceive roles in different domains as requiring them to expend finite resources (e.g., time and energy) in one area that creates a drain in resources in the other domain [38]. Postpartum employees returning to work must also navigate changes in workplace dynamics, including biased expectations. Some coworkers or supervisors may hold gendered stereotypes of postpartum mothers, leading them to be seen as more emotional, fragile, or incompetent upon their return to work [39].

Social support can have a buffering impact though, as higher levels are associated with more positive health correlates and less anxiety among mothers within the first year postpartum [40]. In a sample of 150 married women in the U.S. having their first child and working at least 20 h per week, satisfactory childcare arrangements and supportive relationships with others at work were strong predictors of job satisfaction and greater work-life balance [40]. In a sample of U.S. university employees who were surveyed regarding their work experiences one month after returning to work from maternity leave, those who took longer maternity leaves reported better health status upon their return to work [41].

Empirical evidence is mixed for how new parenthood affects work behaviors and performance. In one sample, pregnant employees in the U.S. perceived declines in their own effort at work, and breastfeeding interference with work indirectly reduced perceptions of daily work progress [42]. On the other hand, pregnant and postpartum employees may feel a need to overperform to compensate for others’ lowered expectations [39]. For example, pregnant workers in the U.S. reported maintaining their work pace and even working harder without accommodations as they tried to maintain a professional image, avoid negative career consequences, and manage work-life conflict [43].

In another study on postpartum mothers who returned to work, Tucker et al. conducted both interviews and surveys with over 200 new mothers in the U.S. at 4, 8, 12, and 16 months postpartum [44]. Survey items and interviews focused on quality of life due to physical health, mental health, and economic conditions. With a focus on the impact of economic hardships during the return-to-work period, the authors emphasized the importance of allowing flexible scheduling and improving support systems at work. In another study focused on women returning to work, interviewers met with participants within the first 12 months postpartum and asked about mental health, social support, work policies, and work-family conflict [45]. Women’s perception of support in the workplace was influenced mainly by the presence of other working parents. Additionally, social support outside of the workplace alleviated work-family conflict. Italian mothers in management positions returning to work emphasized the importance of supervisors conveying the value of work-family policies [46]. As Bourdeau et al. noted, there is an important distinction between the enactment of family-related policies at work, as “enabling” policies allow employees more freedom in determining work conditions, but “enclosing” policies aim to keep employees on site as much as possible and limit flexible work [47].

Upon returning to work, new parents often face challenges related to ongoing sleep disturbances and sleep loss. It is during this period that some postpartum mothers withdraw from their work, with some choosing to leave their jobs shortly after their return. There may be an impact on the time of task for breastfeeding mothers who need to take breaks multiple times throughout the workday, which is described in more detail in the section below. Workplace policies should consider the need for these accommodations, and supervisors should be clear about expectations surrounding them as a sign of the organization’s support of postpartum employees [48]. Although accommodations and flexibility are mandated by law in several countries, enactment of these policies is not always performed consistently or fairly.

It is noteworthy that many of the return-to-work experiences of women after childbirth may persist in the long term and can have far-reaching financial implications if experiences include either interpersonal discrimination or wage-based discrimination. “Motherhood penalties” on wages in one Japanese sample persisted into the mid-career stages for women even 10 years after the birth of a first child [4]. Along with interpersonal and wage-based discrimination at work, postpartum activities such as breastfeeding can also prompt long-term negative attention at work.

### 3.6. Breastfeeding Challenges During Postpartum Return to Work

Women who are returning to work postpartum are often managing breastfeeding challenges, learning to pump and store breastmilk and schedule their work tasks around these activities. This often means they may need to take multiple breaks throughout the day for pumping and storing breastmilk, with each break potentially taking about 30–45 min [49]. The American Academy of Pediatrics recommends exclusively breastfeeding for 6 months and then continuing for at least another 6 months as a supplement to other food [50]. Given that the U.S. does not provide lengthy maternity leave (only 12 weeks of unpaid leave are federally mandated by the Family Medical Leave Act), navigating pumping at work may be an immediate challenge for many postpartum women after only a few months of establishing breastfeeding routines. Even in many European countries with more generous paid maternity leave that lasts many months, returning employees may need to pump and store breastmilk upon their return to work. In 2023, the PUMP Act was signed into legislation in the U.S., providing workplace protections for nursing mothers and requiring organizations to provide reasonable break time from work and a private place to pump for up to one year after the birth of a child [49]. Despite such laws, there is evidence from legal claims in the U.S. that discrimination based on breastfeeding exists and that women working in male-dominated fields are disparately targeted. Law enforcement officers, first responders, and other women in male-dominated jobs account for 43% of breastfeeding discrimination claims.

Postpartum working women also face societal bias and stigmas related to breastfeeding. This stigma may explain why many women stop breastfeeding after returning to work, as indicated by national data in the U.S.; about 60% of infants are breastfed at 6 months, and that percentage falls to about 40% at 12 months [51]. Gabriel et al. conducted two studies of breastfeeding women at work in the U.S. using both interview (Study 1) and daily survey (Study 2) methodologies [42]. Regarding breastfeeding stigma, women reported worrying that breastfeeding and pumping at work would damage their professional reputation and that they were being stereotyped by male coworkers who had negative affective reactions around the pumping and storage process.

Specific incidents of the harassment and discrimination of working women who are breastfeeding and pumping while at work give insight into the range of behaviors working women must cope with daily. Legal claims have included being denied pumping breaks and subsequently experiencing physical pain, lack of privacy for pumping, leaving the employee exposed to clients or to the public in unsafe or unsanitary conditions, and coworkers comparing breastfeeding employees to animals [51]. These legal examples are confirmed by data from Goodman et al.’s interviews with 20 working women who recently had children and reported insufficient time to pump and the lack of an appropriate location for pumping [52].

Groundbreaking studies by Spitzmueller and by Gabriel et al. focused on women’s daily experiences in navigating breastfeeding and work and are particularly noteworthy, as much of their data across several samples used longitudinal and multi-method approaches [42,53]. Gabriel et al.’ studies illustrated that women experience dual affective states that impact breastfeeding goals, work goals, and their sense of work-family balance [42]. They emphasized the importance of these affective pathways in creating both problems and benefits through breastfeeding interference and enrichment and noted that women’s appraisals are critical in labeling experiences as either interference or enrichment. Women who experienced breastfeeding positively reported a better balance between work and family [42]. Breastfeeding compassion, as evidenced by coworkers and supervisors expressing sympathy or understanding and accommodating a need for schedule flexibility, was particularly impactful in boosting women’s daily positive effects. Another study on breastfeeding employees found that most allies providing support did not have direct experience with breastfeeding but instead had been sensitized to challenges around pumping at work, often based on family members’ stories. These allies advocated for better spaces and made sure the organization complied with laws to allow for breaks [54]. Research suggests that increased support such as this throughout the entire postpartum period and not just specific to breastfeeding can have a positive impact on employees.

### 3.7. Impact of Social Support for Pregnant and Postpartum Employees

Social support during both pregnancy and in the postpartum return-to-work phase can be critical to employees’ well-being. A nationally representative sample of over 43,000 Italian women indicated that most women (over 75%) who were employed before the birth of their children remained employed postpartum [55]. Previous seniority and education level, as well as the proximity of the new child’s grandparents, which likely indicated available childcare as well as social support, were strong predictors of women’s returning to work.

Regarding support from those at work, Chawla et al. developed an index of allyship at work for postpartum mothers [56]. They specified five types of postpartum allyship from coworkers and supervisors: providing access to a parent-worker community, co-navigating the HR infrastructure, creating temporal spaces for motherhood, validating the worker’s identity, and validating the parent or mother identity. Notably, promising results related to supervisor support indicated that women perceived supervisors as supportive when they expressed excitement upon their disclosure of the pregnancy and when they spoke about potential accommodations [57]. On the other hand, coworkers’ negative or inconsiderate reactions can exacerbate postpartum depression [58].

Support from other working parents upon the return to work appears particularly important [45]. This could be boosted by work design arrangements, such as providing lactation spaces that allow for semi-private social interactions with other mothers, increasing the likelihood of social support. In a UK sample of women in their second and third trimesters, there was evidence of an ameliorating effect from coworker social support on the relationship between pregnancy-related discrimination and psychological well-being [20]. In the same sample though, having supervisor support did not have a similar impact on well-being. These results emphasize that not all sources of social support at work are the same, and thus it is critical to assess the source of support when using this construct in research studies.

The importance of other similar figures who are perceived as role models by the employee cannot be understated in terms of the successful integration of a new identity based on parenthood with the identity of an employee. In a sample of pregnant Dutch employees, role models were especially crucial during the second trimester and particularly so for women working in male-dominated occupations [27]. When they did not find role models who were perceived as successful in balancing multiple roles, the women interviewed in the study questioned their own competence. Lacking role models, women reported feeling confused and vulnerable, with increased levels of stress and strain [27].

The cognitive framing of returning to work postpartum is also impactful; there are benefits to framing work-related postpartum changes as adaptive changes that can produce boosts in function rather than focusing on a risk of decline in cognitive functioning [17]. This highlights that not only can pregnancy and parenthood impact women’s experiences at work, but the work context itself (and social support, or lack thereof) can affect women’s postpartum transitions back to work. Based on research focused on social support provided to pregnant and postpartum working women, allies are clearly important to postpartum employees’ well-being. These allies may be especially helpful if they are coworkers or supervisors with similar experiences, particularly when it comes to breastfeeding support. Additionally, male supervisors can be helpful allies in prompting organizational accommodations [59]. Regardless of the supervisor’s gender, clear communication and psychological support from supervisors is related to higher postpartum levels of commitment and work engagement, as evidenced by a sample of female managers in Italy [46].

On a larger scale, perceived organizational support (POS) during the postpartum return-to-work phase is critical and is often reflected in supervisors’ actions. Specifically, POS references the extent to which employees feel valued and that their contributions matter. Regarding pregnant and postpartum employees, this support can be demonstrated by access to and ease of use of family-friendly work policies. Notably, the influence of higher levels of POS for a new mother returning to work can even have a positive spillover effect to their partner as well, reducing stress for both [60]. At multiple time points, Little and Masterson sampled new mothers in the U.S. and their partners at the end of the woman’s pregnancy, one week postpartum, and the week the mother returned to work. The perceived organizational support felt by the postpartum mother had the impact of reducing work-family conflict for both partners as well as reducing the partner’s counterproductive work behaviors [60]. Boosting such support had far-reaching impacts, and this should be the goal of organizations, including demonstrating concern for new parents’ well-being. Those authors also cautioned that benevolent sexism should be avoided, and actions such as assigning women fewer challenging tasks upon their return to work under the guise of “being helpful” only signals that their career development has been slowed by their motherhood status [60].

An additional source of support for pregnant and postpartum working women that is extremely understudied is the employee’s health care provider. Providers may act as allies when they provide information about what work accommodations may be available and how to ask for them. They can also offer documentation and notes so that employees can more easily ask for needed accommodations. In interviews with 20 working women who had been pregnant in the past five years and were either low-income, hourly wage workers or working in service or retail jobs, Goodman et al. described how employees were confused regarding how to use paid leave and other benefits and legal protections, including breastfeeding accommodations [52].

## 4. Important Implications and Future Directions

In many of the papers we reviewed, the authors noted a need for additional research using samples of employees in lower-income jobs, part-time work, and precarious work. Most studies focused on the experiences of full-time women who tended to be in mid-to-higher-level positions in non-manual or professional positions. Such jobs may provide a context in which women are more likely to return to work after birth, as they may receive higher wages in these occupations that incentivize employees to continue working relative to lower-paying jobs [60]. Women in higher-status occupations might also have easier access to paying for childcare or may have flexible work conditions in which they can work from home part of the week. Women in public-sector occupations such as education or public administration may also have higher levels of job security in many countries, indicating the importance of gathering data on employees’ experiences across more varied occupational roles with different levels of employment benefits and protections.

The occupational sector and type of work contract are also important to examine, and researchers should aim for variation in these factors. Employees working in occupations or countries with ambiguous maternity leave policies or precarious employment contracts are unlikely to return to work, at least not without a great deal of social and financial support. These types of samples tend to be underrepresented in this research, but precarity of work impacts women’s vulnerability to harassment and discrimination while pregnant, as well as their postpartum return-to-work decisions and experiences.

Other contextual and cultural factors have a large influence on the vulnerability women feel when targeted with pregnancy-related discrimination as well as the postpartum decision to return to work. Researchers in Italy noted that jobs in the public sector have a relatively high level of stability and more protections against employment discrimination, perhaps helping to recruit employees who plan to begin or expand their families while working [55]. On the other hand, part-time workers or those with “atypical contracts” in Italy may not be guaranteed many protections at all, meaning no maternity leave.

Country-level differences in workplace legislation provide additional reasons to carry out more cross-cultural work in this area. For instance, Mongolia does not mandate equal treatment of working women with regard to hiring practices or salaries, allowing discriminatory practices to remain unchallenged [61]. However, legislation does guarantee new mothers an equivalent position upon their return from maternity leave. In the Netherlands, the cultural expectation is that new mothers will transition to part-time work upon the arrival of their first child, leading to stigmatization and negative comments from others if they choose to return to work full-time [61].

Regarding social support, it can be impactful in buffering women’s experiences of many types of harassment and discrimination at work, including pregnancy-related incidents. Similar to sexual harassment, a few studies that assessed women’s reactions to pregnancy-related discrimination noted that rather few women make formal complaints to the organization or file a report or legal claim. This is likely to vary across countries, given differences in legal protections. As with sexual harassment, there are many reasons women may be reluctant to formally report this type of discrimination, including job-related vulnerability, particularly for those working part time or with little job security. Adding to these concerns may be uncertainty regarding the security of their jobs upon returning from maternity leave, as well as the impact on the changing the nature of relationships with coworkers and supervisors.

This is where social support from outside sources as well as trusted coworkers may be especially helpful in reducing the negative impact of being targeted based on one’s pregnancy status. As noted earlier, role models who are other coworkers in similar positions in terms of parenthood may be good mentors and sources of support during both the prenatal and postpartum stages. We encourage supervisors to consider establishing mentoring relationships between pairs or small groups of employees with infants who have recently returned to work, perhaps with a more experienced employee who can speak to challenges and coping strategies acting as a facilitator of the group. As noted by Gabriel et al., it can also be helpful to consider the design of break areas and nursing rooms for breastfeeding mothers in terms of providing both private and communal spaces where employees can share their experiences and offer each other support [42]. Health providers should also be encouraged to support their working pregnant or postpartum patients by proactively discussing how to request work modifications if they are needed and help them complete paperwork, if applicable. Even low-cost steps such as providing information in waiting rooms regarding employees’ rights at work while pregnant or as postpartum employees could help women feel supported and better-informed [52].

## 5. Conclusions

The current state of the research literature and related legal discussions on employees’ experiences of pregnancy-related and postpartum discrimination at work indicates that many gains have been made in recent years in specifying the types of behaviors employees are subjected to during these stages of their work lives. Although illegal in many countries, pregnancy-related discrimination does still occur on a relatively frequent basis, particularly for workers who are in vulnerable positions due to financial concerns, dangerous work, or their minoritized identities. Threats of job loss and refusals to make accommodations for pregnant employees by an organization are instances of discrimination that impact employees’ levels of anxiety and depression. Postpartum, as employees grapple with the transition back to work, instrumental support for both work flexibility and breastfeeding accommodations are critical to ongoing commitment to one’s job and to mental health.

## Data Availability

Not applicable.
